# Post-operative pain after root canal preparation with different apical finishing sizes a triple blinded split mouth clinical trial

**DOI:** 10.1186/s12903-024-04527-9

**Published:** 2024-07-16

**Authors:** Mohamed Medhat Kataia, Engy Medhat Kataia, Hala Fayek Khalil, Mohammed Abou el Seoud

**Affiliations:** 1 Department of Endodontics, Faculty of Dentistry, The British University of Egypt, Cairo, Egypt; 2 Restorative and Dental Materials Department, National Research Centre, Cairo, Egypt

**Keywords:** Modified VAS, Post-operative pain, Apical size, Split-mouth, Triple blinded, E3 azure rotary files, Root canal preparation

## Abstract

**Background:**

This is a triple-blinded, prospective split-mouth clinical trial. It is important to shed light on the effect of different apical preparation sizes regarding postoperative pain within the same patient with the same pulpal histological status. The aim is to compare and evaluate the severity of postoperative pain following apical enlargement with two different sizes after the IBF using the visual analogue scale.

**Methods:**

Fifty “teeth” in 25 patients were assigned into two equal groups (25 per group) using E3 Azure rotary files; Group A was prepared two sizes greater than the Initial binding file (IBF) (the largest K file to bind at the actual working length) mesial canals, which were enlarged to 35#/0.04 and 40#/0.04 for the distal canals. Group B was prepared in three sizes larger than the IBF: 40#/0.04 for mesial canals and 45#/0.04 for the distal canals. On a modified VAS form, patients were questioned to indicate the degree of their pain and assisted in narrating their pain intensity during the following periods: 12, 24, and 72 h, and after a week. VAS data were non-parametric and analyzed using the signed-rank test for intergroup comparisons, Freidman’s test, and the Nemenyi post hoc test for intragroup comparisons. The significance level was set at *p* < 0.05.

**Results:**

showed that regardless of measurement time, enlargement of apical preparation was significantly associated with higher pain scores (*p* < 0.001). Within both groups, there was a significant reduction of measured pain score with time, with values measured after 12 and 24 h being significantly higher than values measured at other intervals (*p* < 0.001) and with values measured after three days being significantly higher than 1-week value (*p* < 0.001).

**Conclusion:**

The size of apical preparation had a significant effect on postoperative pain.

**Trial registration number & date:**

NCT05847738, 08/05/2023.

**Supplementary Information:**

The online version contains supplementary material available at 10.1186/s12903-024-04527-9.

## Background

A usual problem after root canal therapy is postoperative pain. Even though enlarging the canal apically has many advantages biologically, it has also been accompanied by a high percentage of postoperative pain [1].

Postoperative pain has been attributed to insufficient instrumentation, irrigation protocol [2], extrusion of irrigants and/or intracanal medications, trauma, missing canals, discomfort before surgery, periapical pathosis, and debris extruding apically [3].

Other factors that are also thought to be the cause of postoperative pain include instrumentation technique [4] instrument design [5] and the final apical size [6].

Evidence shows that infected debris extruded apically throughout the chemo-mechanical instrumentation is the leading source of pain postoperatively and periapical inflammation [3]. Hence, the amount of debris extruded connected with various instruments and instrumentation techniques is important [7].

All instrumentation techniques cause a varying percentage of debris extrusion apically, even with extreme caution to constrain the preparation to the apical constriction. Nonetheless, most newly introduced (NiTi) rotary files cause minimum extrusion of debris in comparison to the original hand K-files. This could be related to their motion action, screw effect, and the extensive irrigation accompanying the use of these instruments [8].

Some rotational procedures are said to limit debris extrusion more than others [3]. E3 Azure rotary file system (Poldent Co. Ltd., Warsaw, Poland) is used in the crown-down technique, and as claimed by the manufacturer, it has an adjusted S-shaped NiTi file with two cutting edges to ensure effective cutting, upward transportation of debris, and decrease preparation time, resulting in less severity of postoperative pain [9]. Its inactive tip grants safe preparations, minimizing the risk of perforations and zipping. Instruments were fabricated in a way to allowed their use in the three-movement types.

Apical preparation size is a very important variable in the process of root canal instrumentation. There have been many conflicts regarding the proper size and how it affects bacterial reduction, root canal dentine strength in terms of conservation, and even post-operative pain [10, 11]. Post-operative pain as a patient-related outcome with different apical sizes is very important.

At present, the apical size preparation that would cause the least amount of postoperative pain is not universally agreed upon. The impact of apical expansion on postoperative pain has been investigated in a few randomised controlled trials [12, 13].

Since endodontic treatment utilizing E Azure rotary files has no clinical trial available to measure postoperative pain, in this study, postoperative pain after apical enlargement with two sizes after the binding file was evaluated and compared to three sizes of enlargement after the initial binding file.

The hypothesis adopted was that postoperative pain will be greater when we enlarge approximately three sizes after the initial binding file.

## Materials and methods

This is a clinical trial, prospective split-mouth, triple-blinded study.

### Ethical approval

Approval of the study design was provided by the research ethical committee of the British University of Egypt( approval number: 23 − 002, Date: 7/3/2023).

### Clinical trial identifier number

The study protocol was registered at the clinical trials website (https://www.clinicaltrials.gov, with registration number, Identifier: NCT05847738. Participants had their treatment in compliance with the World Medical Association Declaration of Helsinki (2008).

### Participant flow diagram

Figure. 1

**Fig. 1:**
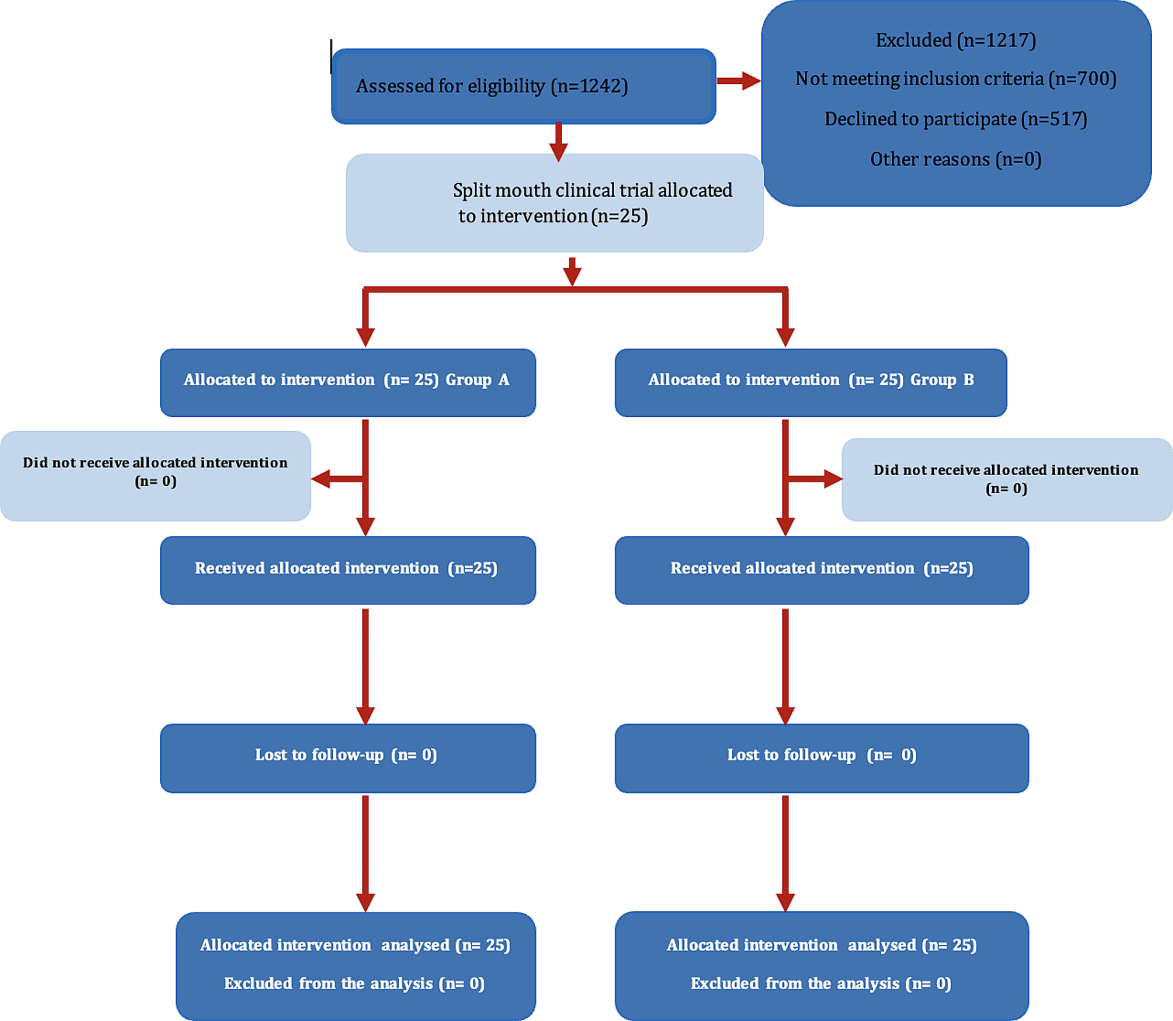
Flow diagram of Patient recruitment

### Sample size calculation

The sample size was calculated via G*Power 3.1.9.4 software concerning data obtained from earlier work (18). For an effect size of 0.97 with an alpha error of 0.05 and a power beta of 0.90, it was calculated that a minimum sample size of 19 participants (sides) per group for a total of 38 sides would be necessary to reach a 95% confidence level of a real difference between the groups. The sample size was raised by 25% to 25 subjects in each group to compensate for drop-outs.

A total of 50 teeth in 25 subjects were selected from walk-in patients presented to the endodontic clinic of the British university in Egypt.

All patients had Bilateral exposed mandibular first or second asymptomatic permanent molars diagnosed with irreversible pulpitis without periapical pathosis.

Patients were chosen according to the inclusion & exclusion criteria mentioned in Table 1.

**Table 1:** Inclusion & Extrusion criteria

Inclusion criteria	Exclusion criteria
Age: 20–40	Age < 20 and > 40
Gender: Males and Females	Pregnancy and nursing
Systematic status: healthy patients (Category: American Society of Anaesthesiologists class 1)	Systematic disease altering the treatment or requiring medications or precautions
Bilateral exposed mandibular first or second permanent molars	Inability to take Ibubrofen
Molars should have separate mesial and distal roots	Symptomatic pulpitis
Mesial roots confirmed to be type IV root canal system (Vertucci)	Widening of periodontal ligaments
Distal root confirmed to be type I root canal system (Vertucci)	Pulp necrosis
Normal periapical radiograph and no bone changes	Periapical abscess
Asymptomatic irreversible pulpitis	Sinus tract
I.B.F. in mesial roots not more than #25, and distal root not more than #30	Not willing to sign the consent
Restorable teeth	Type II root canal system in mesial roots (Vertucci).
Periodontal scoring index < 2	Type II or IV root canal systems in distal roots (Vertucci).
Angle of curvature from 15^0^-25^0^	Angle of curvature more than 25^0^

Pulpal and periradicular condition for all participants was evaluated via thermal tests, palpation, and percussion. The clinical diagnosis of asymptomatic irreversible pulpitis was confirmed when there was an extended response to Green Endo-Ice cold testing (1,1,1,2-tetrafluoromethane; Hygienic Corp., Akron, Ohio, USA) and when deep caries was seen on radiographic view.

Periodontal probing was done. Periapical radiographs were taken by a Paralleled long cone using digital radiography (Kodak RVG 5100, Ontario, Canada).

Cases diagnosed that didn’t conform with the inclusion criteria were referred to the dental clinic of a British university for dental care. Cases that met the criteria were chosen for this study. Participants were verbally briefed about the procedure’s merits, that the treatment results would be used in this study, and the risks of the procedure were explicitly explained to them. After the verbal consent, patients were handed a written consent form to sign (Informed consent in the supplementary files).

**Fig. 2:**
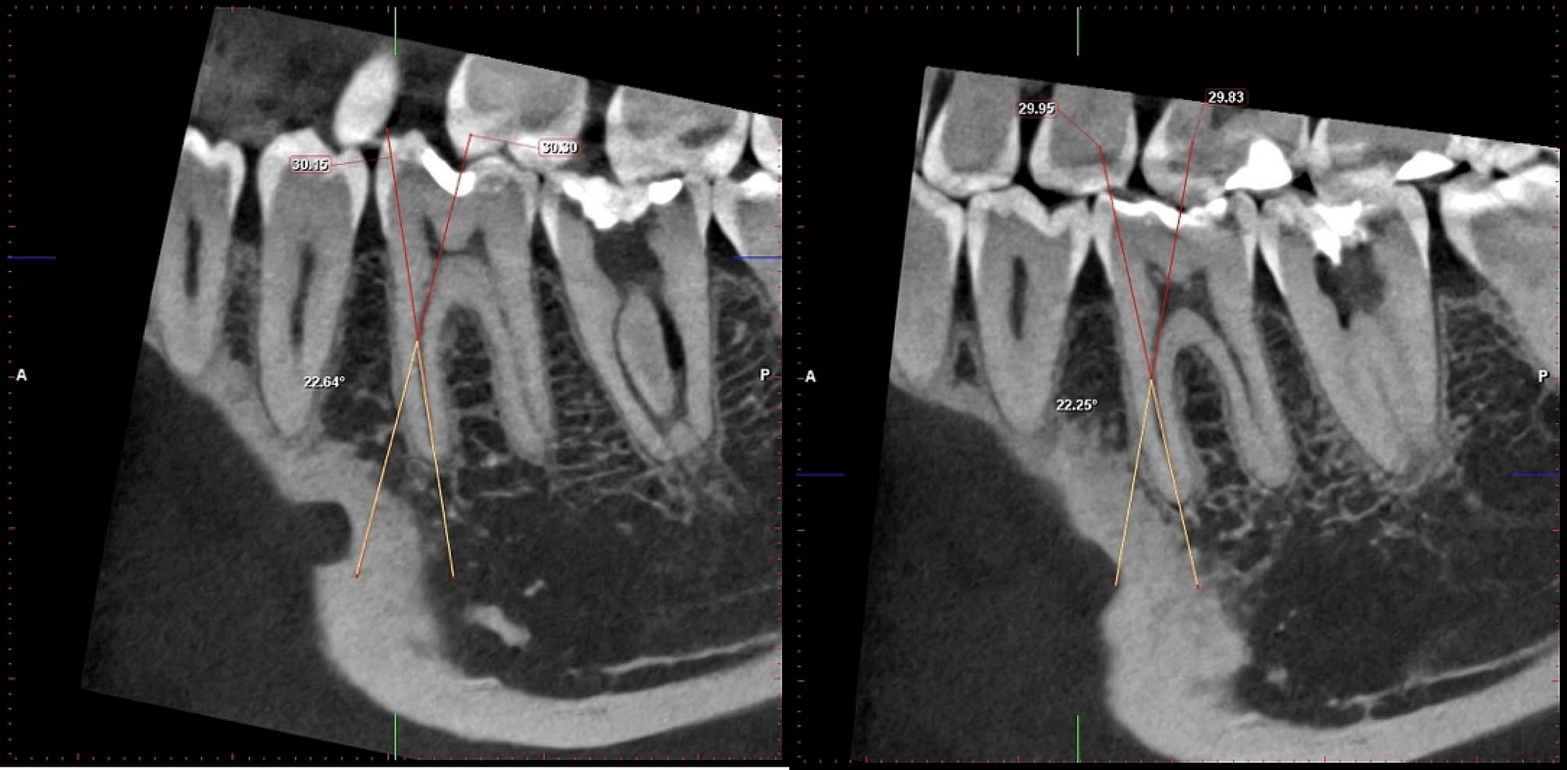
CBCT image showing measuring angle of curvature

All patients were scheduled for CBCT to determine the type of root canal system classification and to measure the root canal curvature according to the Schneider method. Figure (2).

Treatment was scheduled for both sides one week apart; all treatments were initiated for the left side of the patient (group A with two sizes larger than the initial binding file), and patients were monitored for pain results over the week. Patients were treated for the right side in their next appointment, and the same follow-up was recorded.

All the subjects underwent inferior alveolar nerve block anaesthesia and 2% lidocaine containing 1:80000 epinephrine for infiltration of the long buccal. After 15 min, to confirm pulpal anaesthesia, patients were questioned about whether they had lip numbness, and the teeth were re-examined using a comparable cold pulp sensibility test. All patients took a second dose (anaesthetic carpule) to ensure the pulpal depth of anaesthesia.

After access cavities were performed, root canal exploration and scouting were carried out, and root canal systems were confirmed to be the types selected for this study.

Working length determination was postponed until after coronal flaring, as several studies reported a decrease in working length by a fraction of a mm following coronal flaring [14].

Dentsply Maillefer’s #15 K-file, together with an apex locator (J. Morita USA, Inc.‘s Root ZX apex locator, Irvine, California, USA), were used to estimate the working length and digital radiography was used to confirm it [15].

To guarantee that all root canals met the inclusion size requirements for mesial and distal canals, mesial canals with initial binding files size 25 and distal canals with size 30 were chosen for each root canal. After patency and glide paths were performed by K-files up to size #10 (Dentsply Maillefer, Ballaigues, Switzerland), engine-driven canal enlargement was applied using E3 Azure files to the final finishing size according to the grouping.

Group A (left side of the patient) was prepared two sizes larger than the initial binding file IBF to size 35#/0.04 mesial canals and 40#/0.04 distal canals.

Group B (right side of the patient) was prepared three sizes larger than the IBF, to size 40#/0.04 mesial canals and 45#/0.04 distal canals.

Torque and speed were adjusted according to the manufacturer( Speed = 300 rpm, torque = 2 Ncm), the system was employed in a crown-down procedure with the following steps: First, Endostar E3 Azure Small File No. 1 (06/20) orifice was used, file No. 2 (06/25) was then used to shape the canal up to two-thirds of its working length. Using an apex locator and a size 15 hand file, the working length was examined. Then, file number two was presented in its entirety. Using file No. 3 (04/30), the apical portion of the root canal was then shaped to its maximum working length. For group A, apical preparation was carried out until files 35/04 and 40/04 for the mesial and distal canals, respectively. For group B, distal canals were apically prepared to size 45/04, while mesial canals were sized 40/04.

Cleaning of the instrument’s flutes was done after three pecks. 5 mL of an irrigation solution containing 2.5% NaOCl were used for irrigation with a 27-gauge, notched-tip needle (Monoject needle, Tyco Healthcare Group LP, Kendall, Massachusetts) after each instrument. The used needle depth was adjusted by rubber stoppers to be 3 mm shorter than the measured working length. 5 mL saline was used as a final flush.

Finally, canal filling was accomplished using a continuous wave of condensation with a carrier that can fit shorter than the working length by 3 mm [16]. For adequate thermoplasticization of apical gutta-percha, a single cone with matched-size gutta-percha cones and an epoxy resin-based sealer (AH Plus, Dentsply DeTrey, Germany) were used. Finally, the tooth was temporised (Cavit, 3 M ESPE, Germany) to seal the access cavity.

All the steps were carried out by the same clinician to exclude interpersonal variability in the treatment procedures.

To measure postoperative pain, all patients were comprehensively trained on the modified VAS scale by the same instructor [17]. Patients took the VAS form with them. They were asked to note their pain readings at the point that best showed their pain level. So, patients were considered the first blinded, blinded to the intervention. Patients were contacted for assistance in reporting pain on the modified VAS form in the following periods: 12, 24, and 72 h and.

one-week, modified VAS reading was recorded by a second blind clinician (One of the investigators in this research) blinded to the groups. All patients were given a prescription for 600 mg of Ibuprofen twice daily for four days, with instructions to take it only in cases of severe pain that is not tolerable and replaced by other patients; according to this, two patients were excluded and replaced.

Data was sent to the statistician with group names only to fulfil the triple-blinding criteria.

### Statistical analysis

Categorical data were presented as frequency and percentage values. Numerical data was represented as mean, standard deviation (SD), median and interquartile range (IQR) values. They were analyzed for normality by checking data distribution and by using Shapiro-Wilk’s test. mVAS data were non-parametric and were analyzed using a signed rank test for intergroup comparisons and Freidman’s test followed by the Nemenyi post hoc test for intragroup comparisons. The significance level was set at *p* < 0.05 within all tests. Statistical analysis was performed with R statistical analysis software version 4.3.2 for Windows^1^.

## Results

In this study, approval of the clinical trial was cleared on 08/05/2023. Patients in the endodontic clinic, faculty of dentistry, and British University in Egypt were received, and treatment protocols commenced for the participants who met the inclusion criteria, starting 15/05/2023. The number of participants in each group was completed by 28/09/2023. Each member of the participants was followed up routinely after the treatment was carried on individually according to the specific follow-up timetable intervals mentioned in the methodology (12, 24, and 72 h and 1 week), and all follow-up data was collected by the 17th of October, which took five months to complete treatment.

Twenty-five patients were enrolled in this study; all patients had Bilateral exposed mandibular first or second asymptomatic permanent molars with signs of asymptomatic irreversible pulpitis and a normal periapical radiographic image. Among the enlisted patients were 12 males and 13 females; their age was variable from 20 to 40 years, meeting our inclusion criteria. A total of 50 sides were enclosed in the study, 25 per group. Patients who took the prescribed Ibuprofen (*n* = 2) were excluded and replaced by new cases in another interval.

**Fig. 3:**
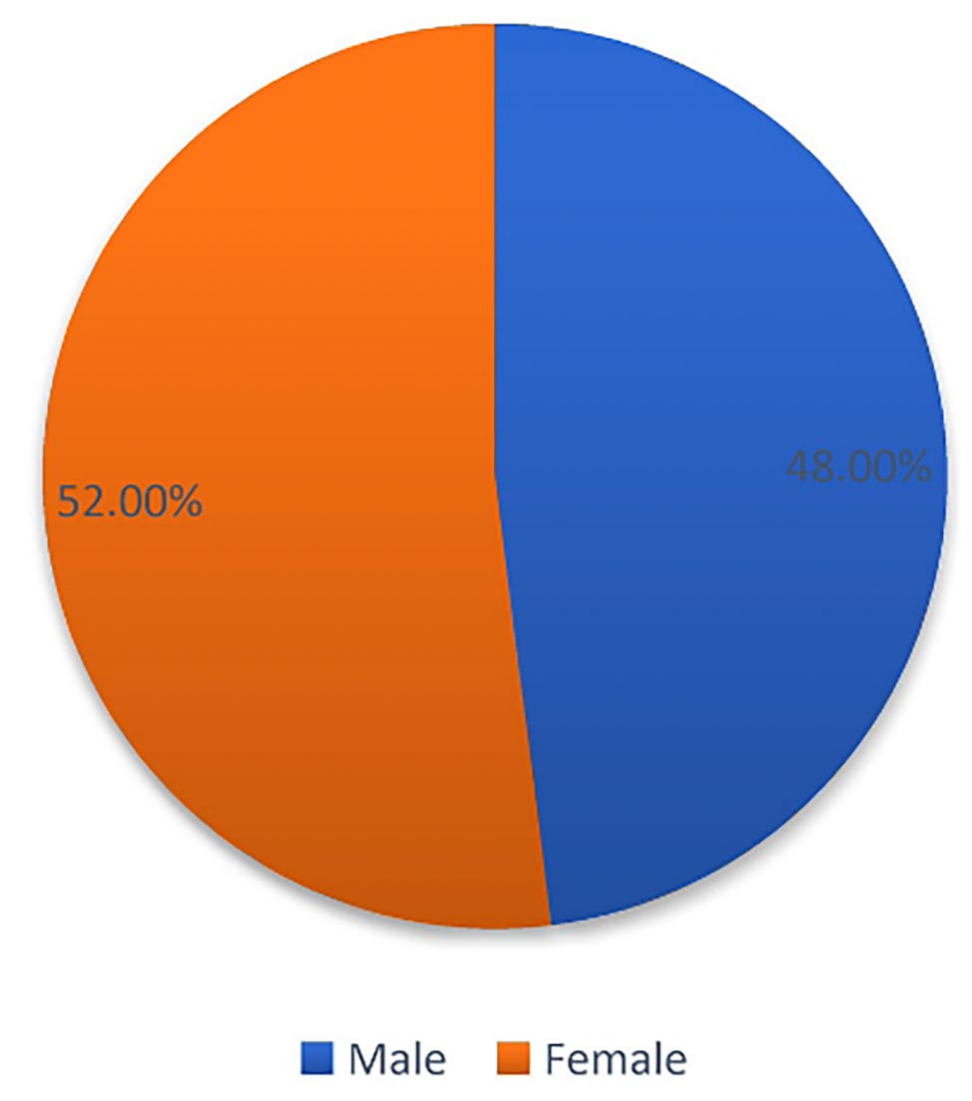
Pie chart showing gender distribution

The test for normality (Shapiro-Wilk’s test) revealed that the data were non parametric or not normally distributed. The split-mouth study was conducted on 25 cases (i.e., 12 males and 13 females) with a mean age of (32.08 ± 4.87) years, which is nearly equal. Gender distribution is presented in Fig. (3).

**Fig. 4:**
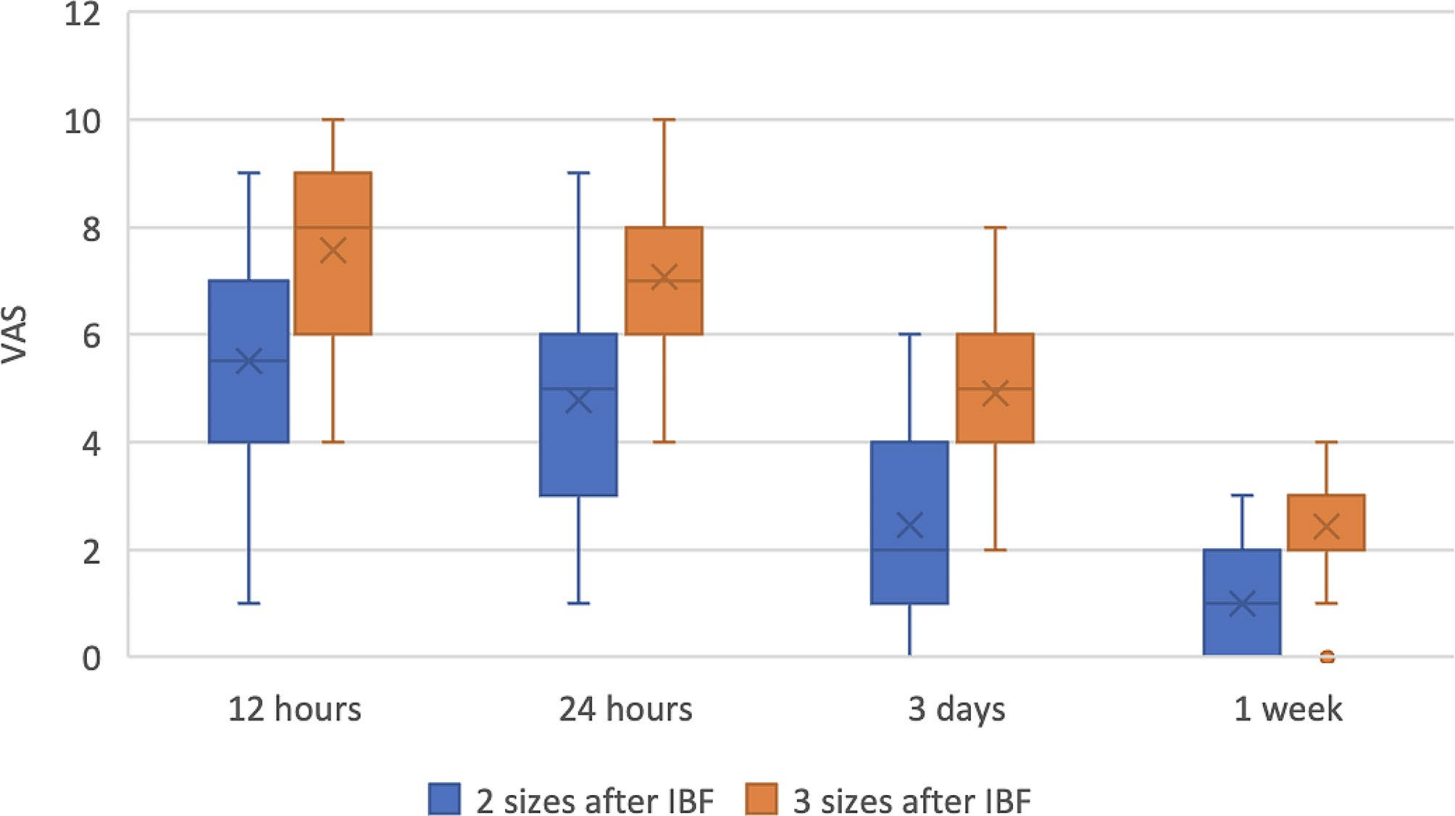
Box plot for VAS values

In Table (2) shows the Summary statistics and results of inter and intragroup comparisons for post-operative pain measurements are presented in Fig. (4). Results showed that regardless of measurement time, enlargement of apical preparation with 3 sizes after IBF was significantly associated with higher median pain scores (V = 0.00, *p* < 0.001). Within 2 sizes after the IBF group (χ^2^ = 135.48, *p* < 0.001) and 2 sizes after the IBF (χ^2^ = 146.05, *p* < 0.001), there was a significant reduction of measured median pain score with time. For both groups, post hoc pairwise comparisons showed median pain scores measured after significantly higher than those measured after 3 days and 1 week (*p* < 0.001). 12 and 24 h were significantly higher than those measured after 3 days and 1 week (*p* < 0.001). In addition, they showed scores measured after 3 days to be significantly higher than 1-week values (*p* < 0.001).

**Table 2:** Summary statistics, inter and intragroup comparisons of mVAS

Interval	Measurement	2 sizes after IBF	3 sizes after IBF	Test statistic	*p*-value
**12 h**	***Mean ± SD***	5.52 ± 2.00^A^	7.58 ± 1.70^A^	**0.00**	< 0.001*
	***Median (IQR)***	5.50 (3.00)^A^	8.00 (3.00)^A^		
**24 h**	***Mean ± SD***	4.78 ± 2.00^A^	7.08 ± 1.58^A^	**0.00**	< 0.001*
	***Median (IQR)***	5.00 (3.00)^A^	7.00 (2.00)^A^		
**3 days**	***Mean ± SD***	2.46 ± 1.79^B^	4.92 ± 1.56^B^	**0.00**	< 0.001*
	***Median (IQR)***	2.00 (3.00)^B^	5.00 (2.00)^B^		
**1 week**	***Mean ± SD***	1.00 ± 0.93^C^	2.42 ± 0.99^C^	**0.00**	< 0.001*
	***Median (IQR)***	1.00 (2.00)^C^	2.00 (1.00)^C^		
**Test statistic**		**135.48**	**146.05**		
**p-value**		< 0.001*	< 0.001*		

SD = Standard deviation, IQR = Interquartile range, Values with **different superscript letters** within the **same vertical column** are significantly different, *Significant (*p* < 0.05).

## Discussion

The present study was designed to evaluate the effect of apical preparation size on postoperative pain.

All patients underwent routine shaping and irrigation procedures for anatomically equivalent teeth with the same diagnosis for uniformity [18]. Mandibular Molars were chosen in this study as it is considered the most affected teeth in the entire dentition [19]. Asymptomatic irreversible pulpitis without periapical pathosis was selected to prevent variabilities among the results.

E3 Azure rotary file system (Poldent Co. Ltd., Warsaw, Poland) is used in the crown-down technique, and as claimed by the manufacturer, it has an adjusted S-shaped NiTi file with two cutting edges to ensure effective cutting, upward transportation of debris, and decrease preparation time, resulting in less severity of postoperative pain [9]. Its inactive tip grants safe preparations, minimizing the risk of perforations and zipping. Instruments were fabricated in a way to allowed their use in the three-movement types.

Regarding number of visits, a Cochrane systematic review reported that there is no evidence to suggest that one treatment approach (single-visit or multiple-visit root canal therapy) is superior to the other or can avoid all short- and long-term issues [20], so according to this study, It was decided to finish the therapy in one visit because it would be more convenient for the patient and the operator in terms of time, especially that there is no periapical pathosis and periodontal issues.

A specific age range (20–40) was selected to limit the difference in pain perception according to age [21].

A paper visual analogue scale (modified VAS) was used in to assess pain intensity [17].

When compared to maxillary posterior teeth (26%), postoperative pain has been reported more frequently for mandibular posterior teeth (42%), likely because of the thick cortical mandibular plate, which permits exudates to build up and increases the intra-periapical pressure that causes discomfort [22]. Hence, our study was conducted on the first and second mandibular molar teeth.

Every individual may vary depending on their response to a painful stimulus. Also, the pain threshold varies from high and low depending on the patient’s type of threshold. Also, we have to keep in mind the extent of the inflammation in the pulp, either coronally or entirely, which also may affect the degree of subsequent pain [22].

On the other hand, pain may be affected by different types of teeth in diverse populations (pain is multifactorial and influenced by factors inherent to patients), different irrigation solutions, different time evaluations and, at last, different types of systems [23].

Postoperative pain after biomechanical preparation using E3 Azure heat-treated files was assessed. The assessment was done at 12, 24 h, after three days, and after one week [24], 12-hour, 24-hour and 72-hour periods were selected because they usually represent the period of the maximum peak of pain [24, 25].

In this study, it was shown that regardless of measurement time, enlargement of apical preparation was significantly associated with higher pain. As the diameter of the apical foramen increases pain increases due to various reasons: An extrusion beyond the apex of chemically active solutions, secondary to debris exhibited pain postoperatively [26], treating vital pulp promotes more intensive secretion of inflammatory mediators, such as prostaglandins, leukotrienes, serotonin, histamine, and bradykinin [27], Any injury to the periapical tissue during RCT promotes more intensive secretion of inflammatory mediators, such as prostaglandins, serotonin, leukotrienes, histamine, and bradykinin, which are considered to be mediators of pain [28]. which comes in agreement with Saini et al. [29]. who reported that enlargement of the apical foramen during root canal treatment increased the incidence and intensity of postoperative pain. Results showed that 12 and 24 h were significantly higher than those measured after 3 days and 1 week (*p* < 0.001). with the maximum VAS mean value at 12- and 24-hours post-treatment for both groups and decreasing with time. Also, 3 days was significantly higher than one week. This comes in agreement with varies studies [12, 30–32], which may be because all patients had the procedure accomplished in a single visit. It was shown that higher post-operative pain incidences were observed after single-visit treatment [23].

Also, a notable decrease in the incidence of postoperative pain was observed. Moreover, after 1 week no patient presented pain and no participants scheduled reintervention during the observation period. Both outcomes are commonly observed in most postoperative pain studies [32, 33].

Another factor that may add to the reported postoperative pain is that the study was carried out on mandibular molars, which is in agreement with a study that showed a greater frequency of post-operative pain with multi-root teeth when compared to single-root teeth [34].

The hypothesis was proven that postoperative pain would be greater when we apically enlarged three sizes after the initial binding file.

The smaller sample size and the determination of apical enlargement size based on the IBF may be considered limitations of this study as CBCT is considered a reliable method for determining the diameter of the apical part of the root canal.

Future research is suggested to compare postoperative pain experienced by patients with symptomatic, asymptomatic, irreversible pulpitis after root canal preparation with E3 Azure rotary instruments.

## Conclusions

- The size of the apical preparation had a significant effect on postoperative pain.
- Variations in studies that have been presented may be due to the subjectivity of a patient’s perception of pain and/or differences in methodology.

### Electronic supplementary material

Below is the link to the electronic supplementary material.

Supplementary Material 1

Supplementary Material 2

Supplementary Material 3

Supplementary Material 4

## Data Availability

Data is provided within the supplementary information files.

## Footnotes

1 R Core Team (2023). R: A language and environment for statistical computing. R Foundation for Statistical Computing, Vienna, Austria. URL https://www.R-project.org/.
