# Supplementary material for: Post-operative pain after root canal preparation with different apical finishing sizes a triple blinded split mouth clinical trial
Source: BMC Oral Health. 2024 Jul 16;24:800. doi: 10.1186/s12903-024-04527-9 (PMC11250953; doi:10.1186/s12903-024-04527-9)
Supplement: Supplementary file 1 — Supplementary Material 1 [file 12903_2024_4527_MOESM1_ESM.docx]

| Total number of patients | 25 |
| --- | --- |
| males | 11 |
| females | 14 |

| Male | Female |
| --- | --- |
| 22 | 28 |
| 33 | 34 |
| 29 | 30 |
| 25 | 25 |
| 31 | 40 |
| 39 | 34 |
| 32 | 31 |
| 38 | 23 |
| 37 | 37 |
| 35 | 32 |
| 34 | 29 |
| 33 | 34 |
|  | 37 |
|  |  |
|  |  |
|  |  |
|  |  |
|  |  |
|  |  |
|  |  |
|  |  |
|  |  |
|  |  |
