# Supplementary material for: Post-operative pain after root canal preparation with different apical finishing sizes a triple blinded split mouth clinical trial
Source: BMC Oral Health. 2024 Jul 16;24:800. doi: 10.1186/s12903-024-04527-9 (PMC11250953; doi:10.1186/s12903-024-04527-9)
Supplement: Supplementary file 2 — Supplementary Material 2 [file 12903_2024_4527_MOESM2_ESM.docx]

RAW DATA:

|  | 12 hrs | | 24 hrs | | 3 days | | 1 week | |
| --- | --- | --- | --- | --- | --- | --- | --- | --- |
|  | 2 sizes after IBF | 3 sizes after IBF | 2 sizes after IBF | 3 sizes after IBF | 2 sizes after IBF | 3 sizes after IBF | 2 sizes after IBF | 3 sizes after IBF |
| 1 | 8 | 10 | 7 | 9 | 5 | 7 | 1 | 3 |
| 2 | 9 | 10 | 8 | 10 | 6 | 8 | 2 | 3 |
| 3 | 7 | 9 | 7 | 9 | 4 | 6 | 1 | 2 |
| 4 | 9 | 10 | 9 | 10 | 6 | 7 | 3 | 4 |
| 5 | 8 | 9 | 8 | 8 | 6 | 6 | 3 | 4 |
| 6 | 7 | 8 | 7 | 8 | 4 | 6 | 2 | 3 |
| 7 | 6 | 7 | 6 | 7 | 3 | 5 | 1 | 3 |
| 8 | 5 | 7 | 4 | 7 | 2 | 4 | 1 | 2 |
| 9 | 7 | 9 | 6 | 8 | 3 | 7 | 1 | 4 |
| 10 | 8 | 10 | 8 | 10 | 4 | 8 | 0 | 3 |
| 11 | 6 | 8 | 6 | 8 | 1 | 6 | 0 | 2 |
| 12 | 9 | 10 | 8 | 9 | 5 | 5 | 2 | 4 |
| 13 | 7 | 8 | 7 | 8 | 5 | 7 | 2 | 3 |
| 14 | 5 | 8 | 5 | 8 | 2 | 6 | 0 | 3 |
| 15 | 4 | 7 | 3 | 6 | 1 | 4 | 0 | 2 |
| 16 | 6 | 8 | 5 | 7 | 2 | 4 | 1 | 3 |
| 17 | 7 | 9 | 7 | 8 | 5 | 6 | 3 | 3 |
| 18 | 6 | 8 | 6 | 7 | 2 | 4 | 1 | 2 |
| 19 | 5 | 7 | 5 | 7 | 2 | 3 | 1 | 1 |
| 20 | 8 | 10 | 7 | 8 | 3 | 7 | 1 | 4 |
| 21 | 3 | 5 | 2 | 5 | 0 | 4 | 0 | 1 |
| 22 | 4 | 6 | 3 | 5 | 1 | 4 | 0 | 2 |
| 23 | 4 | 6 | 4 | 6 | 2 | 4 | 0 | 2 |
| 24 | 5 | 7 | 4 | 6 | 2 | 3 | 1 | 2 |
| 25 | 6 | 8 | 4 | 7 | 3 | 6 | 2 | 4 |
| 26 | 7 | 9 | 6 | 8 | 4 | 5 | 2 | 3 |
| 27 | 4 | 6 | 4 | 6 | 3 | 4 | 2 | 3 |
| 28 | 4 | 6 | 3 | 6 | 1 | 5 | 0 | 3 |
| 29 | 5 | 7 | 3 | 7 | 1 | 4 | 0 | 2 |
| 30 | 6 | 8 | 5 | 8 | 3 | 5 | 1 | 3 |
| 31 | 7 | 9 | 5 | 8 | 2 | 7 | 0 | 4 |
| 32 | 4 | 6 | 2 | 6 | 0 | 4 | 0 | 2 |
| 33 | 2 | 5 | 2 | 5 | 0 | 3 | 0 | 1 |
| 34 | 5 | 7 | 3 | 7 | 1 | 5 | 0 | 2 |
| 35 | 5 | 7 | 5 | 6 | 2 | 4 | 1 | 2 |
| 36 | 6 | 9 | 4 | 8 | 3 | 7 | 2 | 3 |
| 37 | 5 | 7 | 4 | 7 | 2 | 6 | 1 | 2 |
| 38 | 5 | 7 | 3 | 7 | 1 | 5 | 0 | 1 |
| 39 | 4 | 6 | 4 | 6 | 1 | 4 | 0 | 0 |
| 40 | 7 | 9 | 6 | 8 | 5 | 6 | 2 | 3 |
| 41 | 8 | 10 | 6 | 9 | 4 | 6 | 2 | 3 |
| 42 | 2 | 5 | 2 | 4 | 0 | 3 | 0 | 1 |
| 43 | 2 | 5 | 2 | 4 | 0 | 2 | 0 | 1 |
| 44 | 4 | 6 | 3 | 6 | 1 | 3 | 0 | 2 |
| 45 | 5 | 8 | 5 | 7 | 3 | 4 | 1 | 2 |
| 46 | 8 | 10 | 6 | 9 | 4 | 5 | 2 | 3 |
| 47 | 6 | 9 | 5 | 8 | 3 | 5 | 1 | 2 |
| 48 | 2 | 4 | 2 | 4 | 0 | 2 | 1 | 1 |
| 49 | 1 | 4 | 1 | 4 | 0 | 3 | 2 | 2 |
| 50 | 3 | 6 | 2 | 5 | 0 | 2 | 1 | 1 |
| total | 276 | 379 | 239 | 354 | 123 | 246 | 50 | 121 |
| mean | 5.52 | 7.58 | 4.78 | 7.08 | 2.46 | 4.92 | 1 | 2.42 |
