# Supplementary material for: Post-operative pain after root canal preparation with different apical finishing sizes a triple blinded split mouth clinical trial
Source: BMC Oral Health. 2024 Jul 16;24:800. doi: 10.1186/s12903-024-04527-9 (PMC11250953; doi:10.1186/s12903-024-04527-9)
Supplement: Supplementary file 3 — Supplementary Material 3 [file 12903_2024_4527_MOESM3_ESM.docx]

**
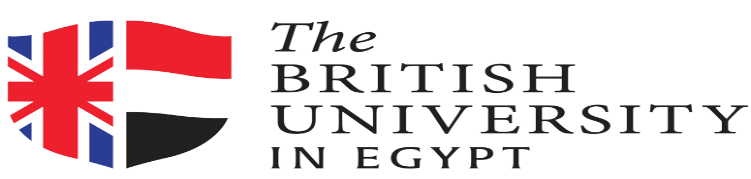
**

**Informed Consent**

**Research Address:**

Triple blinded split mouth clinical trial after single visit root canal treatment to Compare Post-operative pain subsequent to different apical finishing sizes

**Summary of research:**

The root treatment of the patient and the filling of industrial root channels will be done in different sizes and the resulting pain will then be assessed after treatment in the patient.
Lead researcher: a. M. Mohammed Medhat Kataia.

Co-researchers: a. Dr. Engy Medhat Kataia, Dr. Hala Fayek Khalil and Dd. Mohammed Ahmed Omar Abuo el seoud.

**Funding Provider:**

Self-Financing by Researchers.

**General explanation of the situation and what will be done:**

You suffer from severe decay in your teeth that has led to inflammation of the roots and you need to treat roots to clean and fill the canals with a root canal filling.

**Required by the participant in this study:**

If you agree to participate with us in this study, you will be asked to attend one session of the university and we will do root canal treatment steps. After the root canals are cleaned and when they are finished, we will put filling in the roots and evaluate the pain resulting from the filler after 12 hours, 24 hours, 3 days and a week.

**Benefit:**

You will get free root treatment.
Treatment will be provided by specialized doctors.
Treatment will be performed in university clinics with high potential with sterile tools.
Your teeth will be filled with new biomaterials.

**Side effects:**

Symptoms occurring when a root treatment is performed, such as: failure of the procedure, which may necessitate re-treatment, the presence of an edema or pain in the patient, inability to move the jaw for days, breaking tools inside the molar, or a perforation therein, may lead to the removal of the tooth.

**The possibility of accepting or refusing to participate:**

You are never obliged to participate and also have the right to withdraw from the study whenever you wish after informing the responsible operator without your decision affecting the medical care you receive.

**Who will know about your participation in the research:**

The doctors responsible for the research as well as the nurses assisting them and you are free to inform who you want from your family and friends. Information about your condition and treatment will be kept in a safe manner, which is allowed to be consulted only by those responsible for the research.

**If you have any other questions or if you wish to ask any questions during the study.**

You can direct him to Dr. Mohammed Medhat Kataia, Telephone 01004646466

**In case you want to make any complaint during the study.**

You can direct her to Professor Asmaa Yassin, Rapporteur of the Scientific Research Ethics Committee and Professor of the Department of Conservative Therapy at the British University, Telephone: 01001912610

I acknowledge that I have **read, understood and agreed to** participate in the study.

Participant's name:

Phone Number:

Participant's address:

Patient Number Study:

Patient file number: National Number:

Date:

Witness signature: Date:

Signature of researcher: Date:

Ethics Committee Search Number:
