# Supplementary material for: Post-operative pain after root canal preparation with different apical finishing sizes a triple blinded split mouth clinical trial
Source: BMC Oral Health. 2024 Jul 16;24:800. doi: 10.1186/s12903-024-04527-9 (PMC11250953; doi:10.1186/s12903-024-04527-9)
Supplement: Supplementary file 4 — Supplementary Material 4 [file 12903_2024_4527_MOESM4_ESM.docx]

**
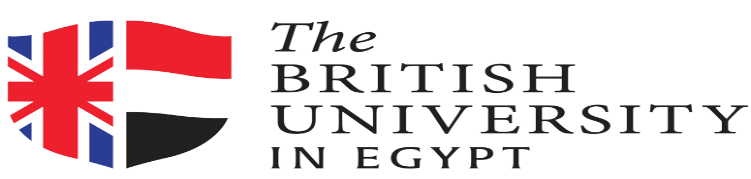
**

**الموافقة المستنيرة**

**عنوان البحث**

تجربة سريرية ثلاثية التعمية للفم المنقسم بعد علاج قناة الجذر في زيارة واحدة لمقارنة الألم بعد العملية الجراحية بعد أحجام التشطيب القمي المختلفة

**ملخص موجز عن البحث:**

سيتم عمل علاج جذور للمريض و حشو القنوات الجذرية بابر صناعيه بأحجام مختلفة وسيتم بعدها تقييم الالم الناتج بعد العلاج عند المريض.

**الباحث الرئيسى:** ا.م محمد مدحت قطايا

**الباحثون المشاركون:** ا.د انجي مدحت قطايا ، ا.م هالة فايق خليل و د. محمد احمد عمر أبو السعود.

**جهة التمويل:** تمويل ذاتي من الباحثين

**شرح عام للحالة وماسيتم اجراءه:**

أنت تعانى من تسوس حاد في اسنانك ادى الى التهاب الجذور و يلزمك علاج جذور لتنظيف القنوات و حشوها باستخدام لواصق نحن نقوم بدراسة وسوف نستعين بانواع لواصق مختلفة لتقييم الالم الناتج بعدها**.**

**المطلوب من المشارك فى هذه الدراسة:**

اذا وافقت على الاشتراك معنا فى هذه الدراسة ،سوف يطلب منك الحضور لجلسة واحدة للجامعه وسوف نقوم بعمل خطوات علاج الجذور و بعد انتهاء تنظيف القنوات الجذرية وعند انتهائها سوف نقوم بوضع حشو في الجذور باستخدام لواصق مختلفة و تقييم الالم الناتج بعد الحشو بعد العلاج ب12 ساعة و 24 ساعة و ٣ أيام و أسبوع.

**الفائدة:** ستحصل على علاج جذور مجاني في الاسنان الذي تعاني من الم فيها .

سيتم تقديم العلاج بواسطة اطباء متخصصين.

العلاج سيتم في عيادات الجامعة ذات الامكانيات العاليه بادوات معقمة .

الحصول على حشو به مواد حيوية جديدة .

**الأعراض الجانبية:** الاعراض الوارد حدوثها عند عمل علاج جذور مثل: فشل الاجراء مما قد يستدعي اعادة العلاج او وجود ورم او الم لدى المريض اوعدم القدرة على تحريك الفك لمدة ايام او كسر ادوات داخل الضرس او حدوث ثقب فيه قد يؤدي الى خلع الضرس.

**إمكانية قبول أو رفض المشاركة:**

**أنت غير مجبر إطلاقا على المشاركة و كذلك من حقك الإنسحاب من الدراسة وقتما تشاء بعد إبلاغ الطبيب المسئول بدون أن يؤثر قرارك على الرعاية الطبية التى تحصل عليها.**

**من سيعلم بمشاركتك فى البحث :**

**الأطباء المسئولون عن البحث و ك****ذلك الممرضات المعاونون لهم و لك مطلق الحرية فى إبلاغ من تريد من أهلك و أصدقاءك كما أن المعلومات الخاصة بحالتك المرضية و علاجك ستكون محفوظة بطريقة آمنة غير مسموح بالإطلاع عليها إلا للمسئولين عن البحث.**

**اذا كانت لديك اي استفسارات اخرى او فى حالة رغبتك فى طرح أى سؤال أثناء الدراسة**

**يمكنك توجيهه إلى الدكتور الباحث الرئيسى الدكتورة محمد مدحت قطايا ، تليفون 01004646466**

**فى حالة رغبتك فى طرح أى شكوى أثناء الدراسة**

**يمكنك توجيهها إلى الأستاذة الدكتورة أسماء ياسين مقررة لجنة أخلاقيات البحث العلمي والأستاذة بقسم العلاج التحفظي بالجامعة البريطانية** **، تليفون:01001912610**

**أقر أنا الموقع أدناه،أننى قد قرأت و فهمت ووافقت على المشاركة فى الدراسة**

| **إسم المشارك:**  **رقم الهاتف :**  **عنوان المشارك:**  **رقم المريض بالدراسة:**  **رقم ملف المريض:** | **الرقم القومى :**  **التاريخ:** |
| --- | --- |
| **توقيع الشاهد:** | **التاريخ:** |
| **توقيع الباحث****:** | **التاريخ:** |

**رقم البحث في لجنة الاخلاقيات :**
